# Altered Nutrient Uptake Causes Mitochondrial Dysfunction in Senescent CD8^+^ EMRA T Cells During Type 2 Diabetes

**DOI:** 10.3389/fragi.2021.681428

**Published:** 2021-08-13

**Authors:** Lauren A. Callender, Elizabeth C. Carroll, Conor Garrod-Ketchley, Johannes Schroth, Jonas Bystrom, Victoria Berryman, Melanie Pattrick, Desiree Campbell-Richards, Gillian A. Hood, Graham A. Hitman, Sarah Finer, Sian M. Henson

**Affiliations:** ^1^ William Harvey Research Institute, Barts and The London School of Medicine and Dentistry, Queen Mary University of London, London, United Kingdom; ^2^ Barts Health NHS Trust, London, United Kingdom; ^3^ Institute of Population Health Sciences, Barts and The London School of Medicine and Dentistry, Queen Mary University of London, London, United Kingdom; ^4^ Blizard Institute, Barts and The London School of Medicine and Dentistry, Queen Mary University of London, London, United Kingdom

**Keywords:** type 2 diabetes, ageing, mitochondria, metabolism, T cell, senescence, inflammation

## Abstract

Mitochondrial health and cellular metabolism can heavily influence the onset of senescence in T cells. CD8^+^ EMRA T cells exhibit mitochondrial dysfunction and alterations to oxidative phosphorylation, however, the metabolic properties of senescent CD8^+^ T cells from people living with type 2 diabetes (T2D) are not known. We show here that mitochondria from T2D CD8^+^ T cells had a higher oxidative capacity together with increased levels of mitochondrial reactive oxgen species (mtROS), compared to age-matched control cells. While fatty acid uptake was increased, fatty acid oxidation was impaired in T2D CD8^+^ EMRA T cells, which also showed an accumulation of lipid droplets and decreased AMPK activity. Increasing glucose and fatty acids in healthy CD8^+^ T cells resulted in increased p-p53 expression and a fragmented mitochondrial morphology, similar to that observed in T2D CD8^+^ EMRA T cells. The resulting mitochondrial changes are likely to have a profound effect on T cell function. Consequently, a better understanding of these metabolic abnormalities is crucial as metabolic manipulation of these cells may restore correct T cell function and help reduce the impact of T cell dysfunction in T2D.

## Introduction

Senescent T cells have been implicated in a number of different chronic inflammatory diseases, such as cancer (Zelle-Rieser et al., 2016), obesity (Shirakawa et al., 2016) and rheumatoid arthritis (Weyand et al., 2014). The responses of these T cells are typically slower and of a lower magnitude than those of healthy individuals; whether the response is measured by proliferation (Goronzy and Weyand, 2017), telomerase activity (Patrick and Weng, 2019) or the induction of signalling events (Mold et al., 2019). This complex immune remodelling also includes the production of an inflammatory secretome called the senescence-associated secretory phenotype (SASP) (Acosta et al., 2013). Phenotypic changes are used to define senescent T cells, typically the loss of the co-stimulatory molecules CD28 and CD27 and increased expression of NK markers such as KLRG1 (Pereira et al., 2020). T EMRA (effector memory CD45RA re-expressing) cells are thought to represent the senescent T cell population, displaying many key features of senescence as well as being implicated in several chronic disease states (Goronzy and Weyand, 2019).

Type 2 Diabetes (T2D) is characterised by progressive peripheral insulin resistance and pancreatic beta cell secretory deficit, together with chronic inflammation and is also more prevalent with increasing age (Palmer et al., 2015). Senescence has previously been implicated in the pathogenesis of T2D. With senescent cell burden being shown to increase in tissues that undergo diabetes-induced damage, such as the kidneys (Docherty et al., 2019) and pancreas (Zhu et al., 2021). Interestingly, CD4^+^CD28^−^ T cells, which are a late stage differentiated population, have been shown to accumulate in T2D (Giubilato et al., 2011). A similar finding was observed in obese mice, where senescent CD4^+^ T cells were found to accumulate in the visceral adipose tissue (VAT) and caused the induction of chronic VAT inflammation (Shirakawa et al., 2016). More recently, CD8^+^CD28^−^CD57^+^ T cells have also been shown to increase the risk of hyperglycaemia in humans, highlighting a potential role for this subset in the pathogenesis of T2D (Lee et al., 2019). Collectively, these studies show an association between T2D and terminally differentiated T cells.

T cells rely on glycolysis to initiate effector function and are extremely sensitive to changes in glucose concentration, hyperglycaemia alone is able to impair T cell activation and migration (Bogdanski et al., 2007; Stegenga et al., 2008). Despite this reliance on glycolysis, mitochondrial function is also important for T cells, playing a crucial role in the oxidative stress response and the provision of key metabolic intermediaries (Desdín-Micó et al., 2018). Alterations in mitochondrial function accompanies numerous chronic inflammatory diseases, and increasing evidence shows that mitochondrial dysfunction contributes to immunosenescence and inflammation (Henson et al., 2014). Both ageing and T2D share important features that include oxidative stress and low-grade inflammation. In ageing, as in the early stages of T2D there is a persistent accumulation of oxidative damage, caused by increased ROS production in all cells (Alonso-Fernández and De la Fuente, 2011). Mitochondria are the main producers and also the main targets of ROS as it damages mtDNA, leading to a vicious cycle of increased ROS production that accelerates both ageing and T2D (Moura et al., 2019). These findings support the notion that impaired T cell metabolism plays a pathogenic role in the development of human T2D.

We explore this idea using functional analysis of mitochondrial metabolism coupled with the assessment of hyperglycaemia and inflammation on mitochondrial fitness. We find that T2D causes significant changes in the metabolism of senescent CD8^+^ T cells: increased lipid storage and mtROS production, together with a reduction in fatty acid oxidation and AMPK activity. Increasing glucose and fatty acid levels in healthy CD8^+^ T cells resulted in a fragmented mitochondrial morphology and increased expression of p-p53, similar to CD8^+^ EMRA T cells from people living with T2D. Furthermore we show that T2D sera contains inflammatory mediators capable of driving an increased rate of CD8^+^ T cell senescence.

## Materials and Methods

### Ethics and Donor Recruitment

Our clinical protocol was approved by the NRES Committee North East (16/NE/0073) and all subjects provided written informed consent. We recruited healthy “young” volunteers (Age range: 20–40 yr, *n* = 18), healthy “old” volunteers (Age range: 52–75 yr, *n* = 30) and people aged >18 yr living with T2D (Age range: 50–77 yr, *n* = 52), identified through the Diabetes Alliance for Research in England (DARE) database, with preferential sampling of people aged 50–80 yr. Exclusion criteria for all participants were: inability to provide written informed consent, infection or immunisation in the month prior to blood collection, any known immunodeficiencies or a history of chemotherapy/radiotherapy, on any immunosuppressive medications within the last 6 mo, significant comorbidity or had a history of neoplasm in the last 10 yr. Peripheral blood was obtained using heparinised tubes, and peripheral blood mononuclear cells (PBMCs) were isolated using Ficoll hypaque (Amersham Biosciences). In addition to a blood sample, the participants age, gender, length of time since T2D diagnosis and medications were documented. However height and weight was not collected (Table 1).

**TABLE 1:** Donor characteristics.

Donor characteristics	T2D participants	Older participants	Young participants
Total number of participants	52	23	25
Age ranges	50–77 yr	49–75 yr	20–36 yr
Median age	64	62	31
Gender (Male	65%	48%	49%
Mean length of time since diagnosis	15 yr	N/A	N/A
Medication(s): 1–2	23%	N/A	N/A
3–6	42%	N/A	N/A
7+	35%	N/A	N/A
Medication category: Diabetes	Metformin, Dulaglutide, Glizcazide, Canagliflozin, Glargine	N/A	N/A
	Humalog, Liraglutide, Gliptin, Levemir		
Statins	Simvastatin, Atrovastatin, Pravastatin		
Other	Rampril, Amlodipine, Bisoprolol, Pregabalin, Morphine		
	Quinninesulphate, Solifenacin, Colecalciferol, Taddalafil		
	Travopost, Doxazosin, Bendro, Citalopram, Fenofibrate		
	Spironolactone, Bendroflumethiazide, Beclomethasone dipropionate, Salamol, Trimethoprim, Paroxetine, Tramadol Isosorbide mononitrate, Omeprazole, Edoxaban, Candesartan		
	Levothyroxine, Tamsulosin, Aspirin		

### Flow Cytometric Analysis and Cell Sorting

Flow cytometric analysis was performed using the following antibodies: anti-glut1 PE (202915) from R&D Systems, anti-KLRG1 PE (MAFA) from Miltenyi Biotec, anti-CD8 PerCP (SK1), anti-CD45RA BV605 (HI100), anti-CD45RA APC (HI1000), anti-CD27 BV421 (O323), anti-CD27 FITC (O323), anti-CD28 BV785 (CD28.2), and anti-CCR7 PECy7 (G043H7) from BioLegend. anti-Ki67 PE (B56; BD Bioscience), anti-p-p53 AF674 (Ser15, 16G8; Cell Signaling) and anti-pAMPK (T172, 40H9; Cell Signaling) were used for intracellular staining using solution AB (ThermoFisher). All samples were analysed using a LSR Fortessa (BD Biosciences) and the resulting data examined using FlowJo software (BD Bioscience). UMAP analysis was performed using FlowJo on down sampled CD8^+^ T cell populations (5,000 events), with standard parameters (nearest neighbours = 15, minimum distance = 0.5) and clustered *via* flowSOM.

Magnetic beads were used to isolate CD8^+^ T cells by positive selection according to the manufacturer’s instructions (Miltenyi Biotec). The purity of T cell subsets was assessed by flow cytometry.

### Mitochondrial Measurements

Mitochondrial mass was assessed by incubating antibody labelled PBMCs with 100 nM of MitoTracker Green FM (ThermoFisher) for 30 min at 37°C, 5% CO_2_. Mitochondrial ROS was measured in labelled PBMCs using 2 µM MitoSOX (ThermoFisher) incubated for 20 min at 37°C, 5% CO_2_. Unfixed samples were immediately analysed using a LSR Fortessa (BD Bioscience).

### Transmission Electron Microscopy

CD27/CD45RA defined CD8^+^ EMRA T cell subsets were isolated and fixed using 2% paraformaldehyde, 1.5% glutaraldehyde in 0.1 m phosphate buffer at pH 7.3. The EMRA T cells were processed as previously described (Henson et al., 2014) and were examined using a Jeol 1010 transmission electron microscope (Jeol) with image capture using a Gatan Orius CCD camera (Gatan).

### Metabolic Assays

Oxygen consumption rates (OCR) and extracellular acidification rates (ECAR) were measured in bead sorted CD8^+^ T cells following 15 min stimulation with 1 μg/ml anti-CD3 and 5 ng/ml IL-2. The assay was performed using RPMI without phenol red and carbonate buffer (Sigma) supplemented with 25 mM glucose, 2 nM L-glutamine and 1 mM pyruvate. The metabolic stress test was performed using 1 µM oligomycin, 1.5 µM fluorocarbonyl cyanide phenylhydrazone (FCCP), 100 nM rotenone and 1 µM antimycin A (Sigma) using a XF-96 Extracellular Flux Analyzer (Agilent).

Fatty acid oxidation (FAO) was performed in substrate limited media: DMEM (Gibco), 0.5 mM glucose, 1 mM L-glutamine, 0.5 mM carnitine, and 1% FCS). 45 min prior to the assay cells were transferred to warm Krebs-Henseleit KH Buffer (KHB) (111 mM NaCL, 4.7 mM KCL, 1.25 mM CaCl2, 2 mM MgSO4, 1.2 mM NaH2PO4) supplemented with 2.5 mM glucose, 0.5 mM carnitine, 5 mM HEPES, pH 7.4. Subsequently, 15 min prior to the assay, 40 μM Etoximir was added to selected groups. Finally, the cells were provided with Palmitate:BSA (Aglient) as a substrate and subjected to a Mitochondrial Stress Test as described above.

### Nutrient Uptake Experiments

Lipid uptake was measured in PBMCs that had been stimulated for 1 h at 37°C with 1 μg/μl of anti-CD3 in complete RPMI-1640 medium. 1 nM of the fluorescent palmitate analogue BODIPY FL C16 (Invitrogen) was then added and the cells incubated in the dark for 10 min. Cells were subjected to surface staining prior to flow cytometric analysis.

### Sera Culture Experiments

PBMCs isolated from a young donor were cultured in either low (5 mM) or high glucose (25 mM) RPMI-1640 medium with or without 10% sera from either T2D patients or healthy age-matched control participants. Antibody staining was carried out on samples at day 0, 3 and 7 and the cells analysed by flow cytometry in order to phenotype the samples.

### Cytokine Array

The Proteome Profiler Human Cytokine Array kit (R&D Systems) was used to quantitate the cytokines present in sera taken from people living with T2D or age-matched individuals according to the manufacturer’s instructions.

### Confocal Microscopy

To assess mitochondrial morphology, CD8^+^ T cells were incubated in either high (25 mM), low glucose (5 mM) or with 200 μM palmitate overnight prior to plating onto 12 well microscope slides at a density of 0.05 × 10^6^ per well. Cells were fixed in 2% PFA and 100 nM of MitoTracker Green FM (ThermoFisher) added for a duration of 30 min at 37°C, 5% CO_2_. 4ʹ,6-diamidino-2-phenylindole (DAPI) (ThermoFisher Scientific) was then added for 10 min at room temperature in the dark. Samples were imaged on a Zeiss LSM 880 confocal microscope with a ×63 oil immersion objective lens. Excitation was at 488 nm from an argon-ion laser. Fluorescence detection was in the green, 488 nm and UV, 405 nm channel.

### Cellular Senescence RT2 Profiler PCR Arrays

Unstimulated CD8^+^ EMRA T cells from T2D and healthy age-matched control participants were isolated using MACS sorting for CD8^+^ T cells and then FACS sorted using anti-CD27/anti-CD45RA for EMRA isolation. RNA was extracted using the RNAeasy Micro Kit (Qiagen) and pre-amplified using the RT2 PreAMP cDNA Synthesis Kit (Qiagen) and RT2 PreAMP Pathway Primer Mix (Qiagen). Resulting gene-specific cDNA was then analysed using the Cellular Senescence RT2 Profiler PCR Arrays according to the manufactures instructions (Qiagen).

### qPCR of Mitochondrial Fission and Fusion Markers

RNA from sorted CD27/CD45RA defined CD8^+^ EMRA T cells was isolated using the RNeasy kit (Qiagen) according the manufactures instructions. Transcripts were quantified using the High-Capacity cDNA Reverse Transcription Kit (Applied Biosystems) and the SsoAdvanced Universal SYBR Green Supermix (Bio-Rd Laboratories) according the manufactures instructions. The following primers were purchased from IDT. Fis1 F: GAT GAC ATC CGT AAA GGC ATC G; Fis 1 R: AGA AGA CGT AAT CCC GCT GTT, Mff F: ACT GAA GGC ATT AGT CAG CGA; Mff R: TCC TGC TAC AAC AAT CCT CTC C, DNM1L F: GGT GAA CCC GTG GAT GAT AAA; DNM1L R: CCT CAG GCA CAA ATA AAG CAG, BActin F: CAC CAT TGG CAA TGA GCG GTT C; BActin R: AGG TCT TTG CGG ATG TCC ACG T.

### Statistical Analysis

GraphPad Prism was used to perform statistical analysis. Statistical significance was evaluated using the paired Student *t*-test or a two-way ANOVA with Bonferroni correction used for post-hoc testing. Data was expressed as mean ± SEM and *p* values are represented using the following notation: p* = <0.05, p** = <0.01, p*** = <0.005 and p**** = < 0.001.

## Results

### CD8^+^ T Cells Display a Premature Aged Phenotype in T2D

**FIGURE 1:**
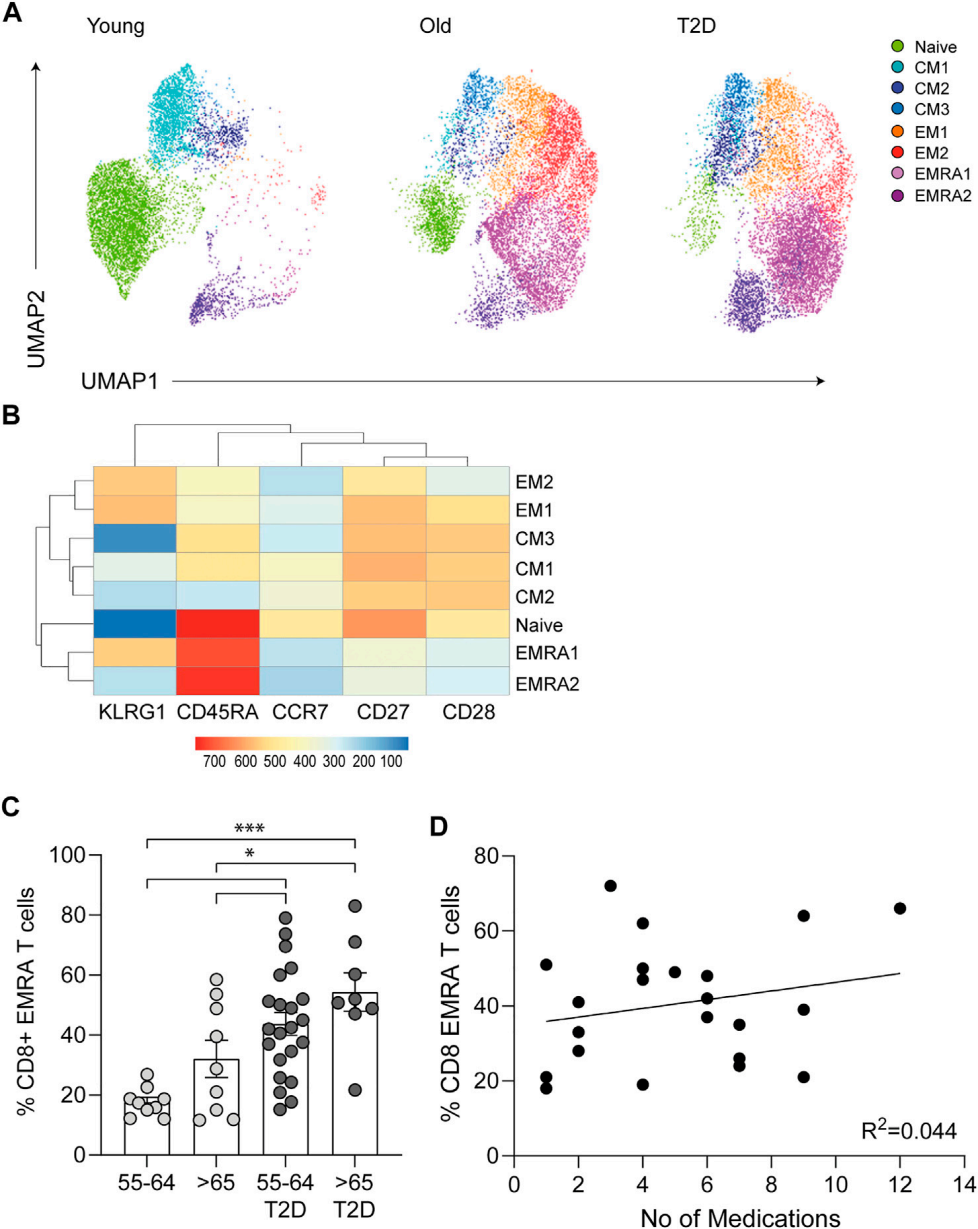
Phenotypic characteristics of CD8^+^ T cells taken from young, older and people living with T2D. **(A)** UMAP dimensionality reduction and clustering of CD8^+^ T cells using CD27, CD28, CD45RA, CCR7 and KLRG1, *n* = 6. **(B)** Heatmap to show protein levels of cell surface markers CD27, CD28, CD45RA, CCR7 and KLRG1 in each cluster, *n* = 6. **(C)** Percentage of CD27/CD45RA defined CD8^+^ EMRA T cells stratified according to age and disease status. Data expressed as mean ± SEM. *p* values were determined using a one-way ANOVA with Bonferroni analysis used for *post-hoc* testing, p* = < 0.05, p*** = < 0.001. **(D)** The relationship between T2D CD8^+^ EMRA T cells and medication shown as CD8^+^ EMRA T cells percentage of the total CD8^+^ T cell population against the total number of medications, *n* = 22. Line of best fit generated using linear regression.

The cell surface markers CD45RA, CD28, CD27, CCR7 and KLRG1 were used to quantify the frequency of CD8^+^ T cell subsets in people living with T2D compared with healthy age-matched controls and young participants. Four populations of T cells can be distinguished using these markers, Naïve T cells (N: CD45RA^+^CD28^+^CD27^+^CCR7^+^KLRG1^low^), central memory cells (CM: CD45RA^−^CD28^+^CD27^+^CCR7^+^KLRG1^low^), effector memory cells (EM: CD45RA^−^CD28^−^CD27^−^CCR7^−^KLRG1^high^) and effector memory CD45RA re-expressing T cell (EMRA: CD45RA^+^CD28^−^CD27^−^CCR7^−^KLRG1^high^). UMAP (Uniform manifold Approximation and Projection) analysis was subsequently performed (Figure 1A). UMAP is an unbiased manifold learning technique for dimension reduction. The plot represents each CD8^+^ T cell subset as a point, which when combined with heat map expression data allows the properties of each cluster to be determined (Figure 1B). UMAP analysis indicate that older people living with T2D have an elevated expression of the senescent EMRA T cell subset. This was confirmed when the data was stratified into people aged 55–64 and those over 65 yr together with disease status. T2D participants were found to have an increased percentage of CD8^+^ EMRA T cells compared to age matched individuals, demonstrating that people living with T2D have a prematurely aged T cell phenotype (Figure 1C, Supplementary Figure S1A). However we did not measure CMV status of the participants, it has been reported that CMV seropositive oldest old (>85 yr) individuals were more likely to have T2D (Chen et al., 2012a). Therefore, any effect CMV may be having on T cell subset profile cannot be excluded.

All people living with T2D were on medication, with the majority taking three to six different types of medication to control their condition (Table 1). To assess the potential impact of medications on CD8^+^ EMRA numbers, we compared the percentages of the T cells with the total number of drugs used, however no relationship was found (Figure 1D). This led us to conclude that the rise in CD8^+^ EMRA T cells was a consequence of T2D and not the medications taken by the participants.

### T2D Alters the Mitochondrial and Metabolic Properties of CD8^+^ EMRAs

**FIGURE 2:**
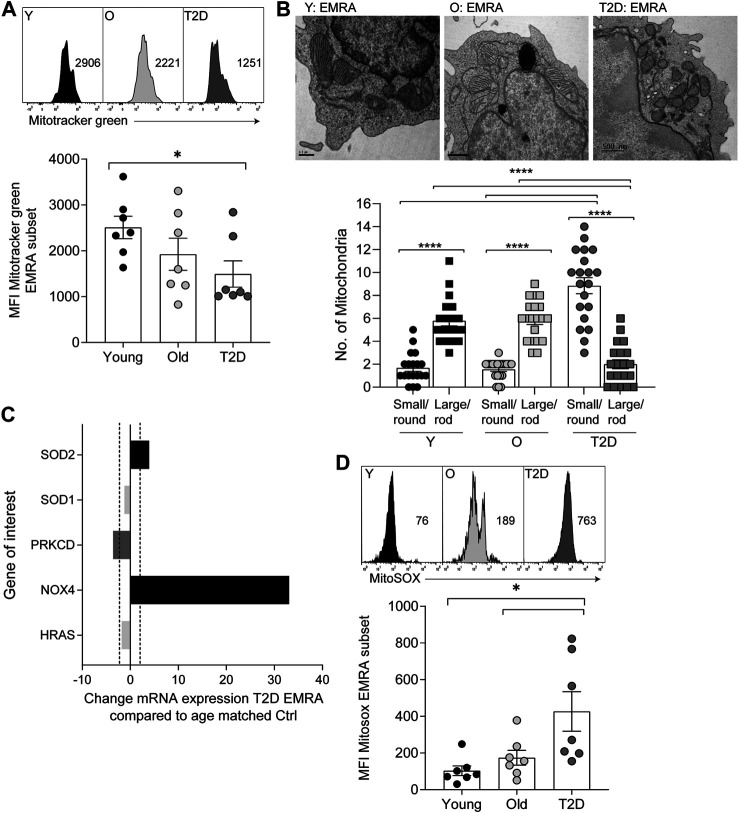
Mitochondrial changes to CD8^+^ EMRA T cells from people living with T2D. **(A)** Representative histograms and quantification of Mitotracker Green in CD8^+^ T cell EMRAs isolated from young, older individuals and people living with T2D. Data expressed as mean ± SEM, *n* = 7. **(B)** Electron microscope images of CD8^+^ EMRA T cells from young, older individuals and people living with T2D, scale bars measure 0.5 µM. **(C)** Cellular senescence RT2 profiler array data showing the change in mRNA expression in genes associated with oxidative stress from T2D CD8^+^ EMRA T cells compared to age matched individuals. Fold change cut-off < 2, *n* = 3. **(D)** Graphs and quantification of MitoSOX staining in CD8^+^ T cell EMRAs isolated from young, older individuals and people living with T2D. Data expressed as mean ± SEM, *n* = 7. *p* values were determined using a two-way ANOVA with Bonferroni analysis used for *post-hoc* testing, p* = < 0.05.

Mitochondrial health and cellular metabolism can influence the onset of senescence in T cells (Callender et al., 2020). CD8^+^ EMRA T cells exhibit mitochondrial dysfunction and impaired OXPHOS when compared to the other memory subsets (Henson et al., 2014). However, the metabolic properties of CD8^+^ EMRA T cells from people living with T2D have not been assessed. The mitochondrial mass of CD8^+^ T cell subsets was determined using Mitotracker green staining. While T2D CD8^+^ EMRA T cells showed a tendency towards a decreased mitochondrial content it was not significantly different from the age-matched controls, however there was a loss of mitochondria when T2D EMRAs were compared to those from young individuals (Figure 2A). When the mitochondria were observed by electron microscopy, mitochondria from T2D CD8^+^ EMRA cells were found to exhibit a different morphology compared to participants without T2D. The mitochondria from participants with T2D were typically smaller, rounder with little or no cristae (Figure 2B). Mitochondria frequently change shape when stressed and fragmented mitochondria can increase reactive oxygen species (ROS) production leading to further damage (Yu et al., 2006).

Next, we went on to assess whether mitochondria from T2D EMRAs had a propensity to produce more ROS than EMRA T cells from healthy individuals. Data obtained from a cellular senescence RT2 profiler array indicated that NADPH oxidases 4 (NOX4) was greatly upregulated in the T2D CD8^+^ EMRA T cells compared with the age-matched control EMRAs (Figure 2C). NOX enzymes, including NOX4, are major contributors to cellular ROS (Chen et al., 2012b). The mitochondrial gene; superoxide dismutase 2 (SOD2), which converts superoxide by-products of OXPHOS into hydrogen peroxide and diatomic oxygen (Velarde et al., 2012), was also elevated. This is likely an attempt to reduce the damaging effects of elevated oxidative stress. Despite the elevated SOD2 expression, examination of mtROS levels using the mitochondrial superoxide indicator MitoSOX revealed that mtROS levels were highest in T2D EMRA subsets compared with the age-matched controls or EMRAs isolated from younger individuals (Figure 2D). These data suggest that the elevated ROS content potentially has a damaging effect of the EMRA T cells from people living with T2D.

### T2D CD8^+^ EMRA T Cells Have an Altered Bioenergetic Profile

**FIGURE 3:**
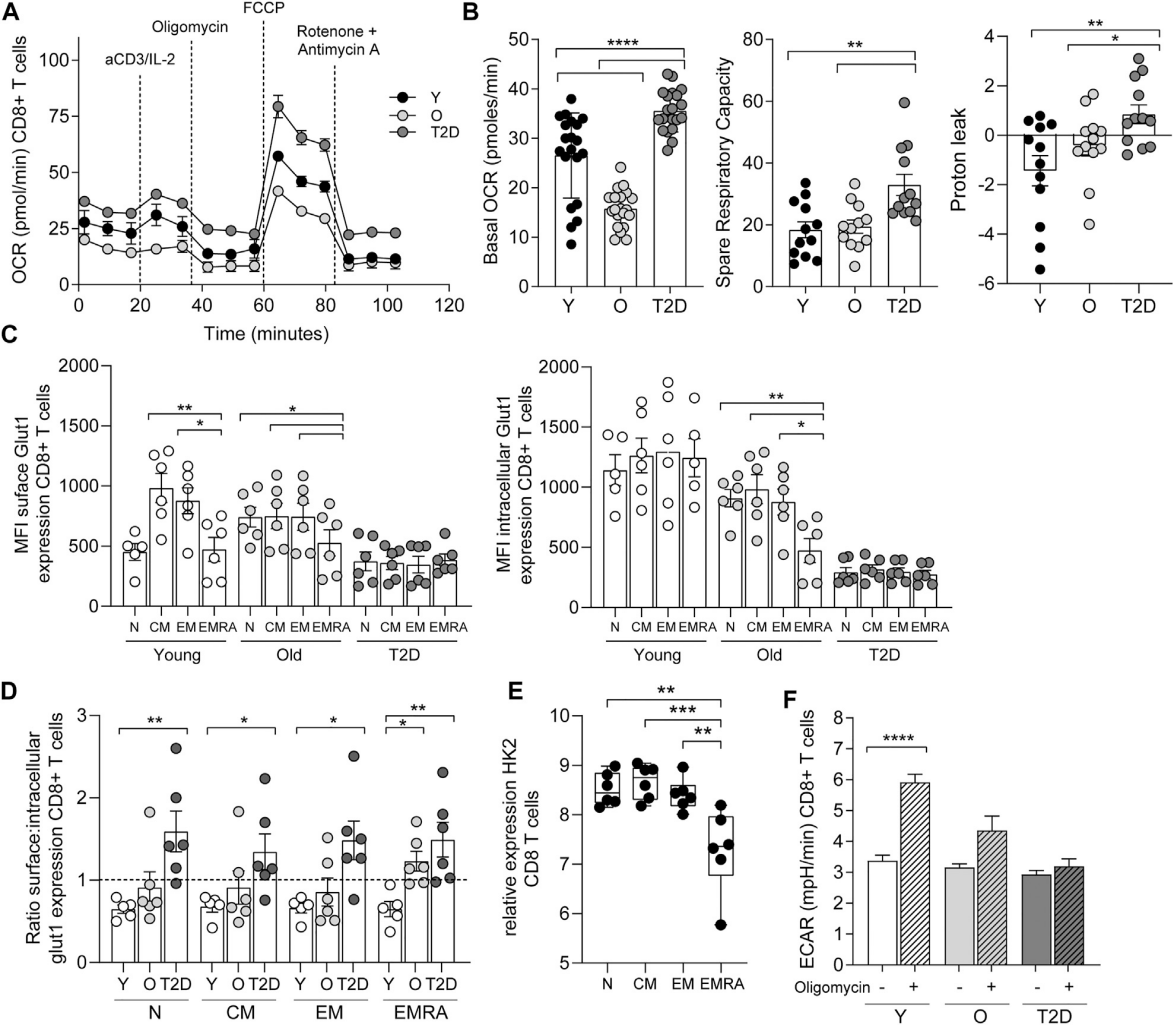
CD8^+^ T cells from people living with T2D show increased oxidative metabolism. **(A)** OCR of the CD8^+^ T cells was measured after 15-min stimulation with 0.5 μg/ml anti-CD3 and 5 ng/ml IL-2, cells were subjected to a mitochondrial stress test using the indicated mitochondrial inhibitors. *n* = 4. **(B)** Basal and maximal OCR along with proton leak following 15-min stimulation with 0.5 μg/ml anti-CD3 and 5 ng/ml IL-2. Data expressed as mean ± SEM, *n* = 4. **(C)** Surface and intracellular Glut1 expression following an 8 h stimulation with 5 μg/ml anti-CD3 in CD45RA/CD27 defined CD8^+^ T cells. Data expressed as mean ± SEM, *n* = 6. **(D)**. Ratio of extracellular/intracellular Glut1 expression. **(E)** Relative expression of hexokinase using the deposited data set GEO Series accession number GSE98640 from six old individuals. **(F)** Extracellular acidification rate (ECAR) following the addition of oligomycin. Data expressed as mean ± SEM *n* = 4. *p* values were determined using a two-way ANOVA with Bonferroni analysis used for *post-hoc* testing, p* = < 0.05, p** = < 0.01, p**** = < 0.001.

The consequence of a reduced mitochondrial content in CD8^+^ EMRA T cells isolated from people living with T2D was investigated further. Due to the limited cell numbers obtained from T2D blood donations we used whole CD8^+^ T cells to investigate metabolic changes. We believe this to reflect the EMRA population as more than 50% of the total CD8^+^ population isolated from people living with T2D are EMRAs (Figure 1C, Supplementary Figure S1A). Furthermore, mitotracker and mitoSOX data show very similar staining patterns when the EMRA subset from young, old and old T2D individuals were compared to the total CD8^+^ fraction (Supplementary Figure S1B). Therefore, we compared the bioenergetic profiles of whole CD8^+^ T cells from young, healthy age-matched controls and T2D participants (Figure 3A). Interestingly, CD8^+^ T cells from people living with T2D had an elevated basal and spare respiratory capacity (Figure 3B) compared to age-matched or younger individuals. We do not believe this to be a consequence of metformin usage, which has been demonstrated to increase oxidative capacity in mitochondria (Wang et al., 2019), as we observed similar flux profiles when we separated the data into those who were and were not treated with metformin (Supplementary Figure S1C). However, despite this apparent increase in respiratory function, CD8^+^ T cells from people living with T2D displayed a far higher proton leak indicative of mitochondrial damage (Figure 3B).

The energy source fuelling the increased oxidative respiration was then investigated. We have shown previously that CD8^+^ EMRA T cells taken from people living with T2D displayed a lack of glucose uptake (Lau et al., 2019). This was confirmed when we investigated the expression of the glucose transporter Glut1. CD8^+^ T cells from old and T2D individuals were found to display a recycling defect, while this prolonged surface duration of the Glut1 transporter we failed to observe an increase in glucose uptake and glycolysis. The rate of nutrient uptake is largely determined by the permanence of transporters at the cell surface, controlled by the balance of endocytosis and recycling (Antonescu et al., 2014). We have shown previously that CD8^+^ EMRAs exhibit defective autophagy (Henson et al., 2014), which promotes Glut1 trafficking (Roy et al., 2017). Together with a lack AKT and mTOR phosphorylation (Henson et al., 2014; Plunkett et al., 2007), both of which control Glut1 recycling. EMRA T cells also show defective endocytosis of Glut1, demonstrated by an increased expression of thioredoxin-interacting protein (TXNIP), which has an inhibitory effect on Glut1 internalisation (Supplementary Figure S1C) (Antonescu et al., 2014). These alterations to receptor recycling resulted in differing levels of surface and intracellular Glut1 expression following an 8 h stimulation with anti-CD3 (Figure 3C). This led to CD8^+^ T cell subsets isolated from young individuals having a surface/intracellular ratio of less than one. Whereas the ratio of EMRA subset from old individuals together with all subsets from people living with T2D was above one, suggesting that Glut1 was retained on the cell surface (Figure 3D). However, despite this we did not find an increase in glycolysis, shown by a drop in hexokinase two gene expression from CD8^+^ T cells isolated from old individuals (Figure 3E). Furthermore, CD8^+^ T cells isolated from old and T2D individuals failed to switch to glycolysis, the extracellular acidification rate (ECAR) with the addition of oligomycin did not increase as seen in the young cohort (Figure 3F).

**FIGURE 4:**
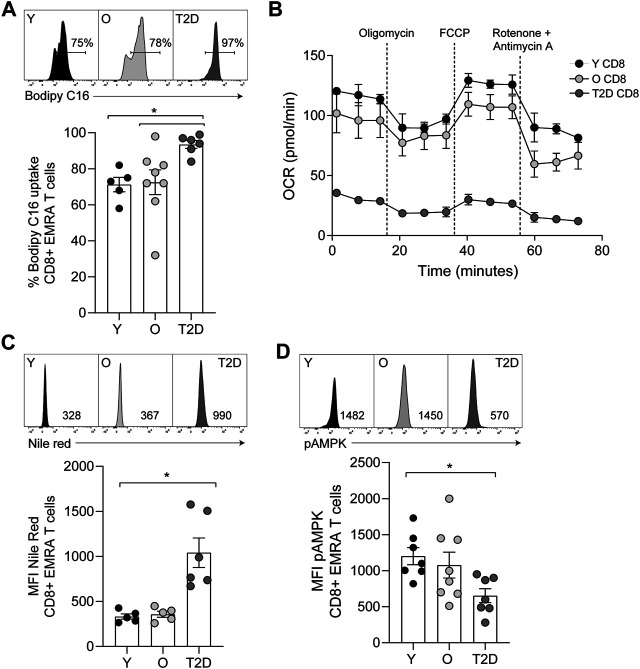
CD8^+^ T cells from people living with T2D show increased lipid uptake but decreased fatty acid oxidation. **(A)** Lipid uptake assessed using BODIPY C16. Representative dot plots and cumulative data. Data expressed as mean ± SEM, young *n* = 5, old *n* = 8, T2D *n* = 6. **(B)** FAO determined OCR of the CD8^+^ T cells was measured after 15-min stimulation with 0.5 μg/ml anti-CD3 and 5 ng/ml IL-2 and incubation with palmitate. Cells were subjected to a mitochondrial stress test using indicated mitochondrial inhibitors. *n* = 2. **(C)** Plots and quantification of lipid droplets visualised using Nile Red in young, older individuals and T2D CD8^+^ EMRAs. Data expressed as mean ± SEM, young *n* = 5, control *n* = 5, T2D *n* = 6. **(D)** Flow cytometry plots and cumulative data of pAMPK in young, control and T2D CD8^+^ EMRAs. Data expressed as mean ± SEM, young *n* = 7, old *n* = 8, T2D *n* = 7. *p* values were determined using a two-way ANOVA with Bonferroni analysis used for *post-hoc* testing, p* = < 0.05.

In contrast to glucose uptake, CD8^+^ EMRA T cells from T2D participants significantly increased uptake of the fatty acid palmitate (Figure 4A). Consequently, we used the Seahorse XF Palmitate assay to investigate fatty acid oxidation (FAO). A significant decrease in FAO was observed in CD8^+^ T cells taken from people living with T2D (Figure 4B). As palmitate was not being utilised, we subsequently used Nile Red to assess the accumulation of lipid droplets. This revealed that CD8^+^ EMRA T cells from T2D participants were indeed accumulating lipid (Figure 4C, Supplementary Figure S1E). Unfortunately, these experiments were unable to determine what energy source was being utilised for mitochondrial respiration by the EMRA T cells from people living with T2D (Figure 3A).

The observed alterations in nutrient usage in T2D CD8^+^ EMRA T cells led us to examine the levels of phosphorylated AMP-activated protein kinase (pAMPK), a master regulator of both glucose and lipid metabolism (Hardie et al., 2012). pAMPK was found to be reduced in the T2D CD8^+^ EMRA T cells compared to EMRAs from people without a diagnosis of T2D (Figure 4D, Supplementary Figure S1E). This finding supports our observation that the data is not an artefact of metformin usage, which activates AMPK by increasing its phosphorylation at Thr-172 (Lin and Hardie, 2018). These data suggest that the change in nutrient uptake caused by a failure to phosphorylate AMPK drives metabolic collapse in CD8^+^ EMRA T cells.

### Inflammatory Factors Together with High Serum Glucose Induce a Senescent Phenotype

**FIGURE 5:**
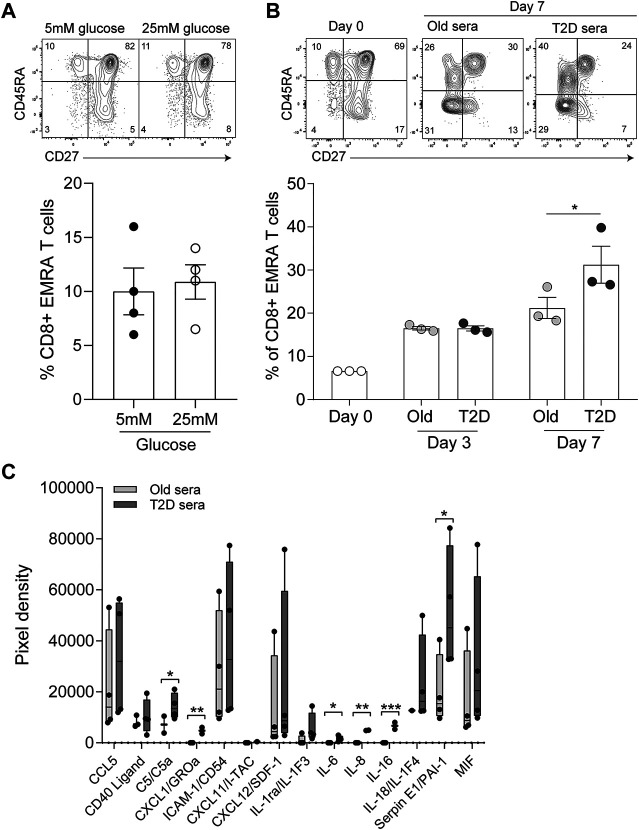
Inflammatory mediators in T2D sera drives the senescent phenotype. **(A)** Plots and quantification of CD8^+^ EMRA T cells following 7 days in culture with 10% FCS and either low (5 mM) and high (25 mM) glucose. Data expressed as mean ± SEM, *n* = 4. **(B)** Plots and quantification of CD8^+^ EMRA T cells at day 0, 3 and 7 of culture with 10% sera from age-matched control and T2D participants. Data expressed as mean ± SEM, *n* = 3. **(C)** Cytokine levels quantified from a cytokine array using T2D serum or sera from age matched controls. *n* = 4. *p* value were determined using a paired *t*-test, p* = < 0.05, p** = < 0.01, p*** = < 0.005.

Finally, we assessed whether the senescent phenotype was induced by high concentrations of glucose or by inflammatory mediators present in the sera of those with T2D. To mimic the environment of T2D we first cultured young PBMCs in either low (5 mM) or high glucose (25 mM), however we found no difference in the percentage of CD8^+^ EMRA T cells observed between the two conditions (Figure 5A). Then we cultured young PBMCs with either 10% T2D or age-matched control sera. We found no difference in CD8^+^ T cell phenotype between the two conditions at day 3. However, by day 7 the T2D sera resulted in an increase in the CD8^+^ EMRA T cell population (Figure 5B). We then went on to determine the factors present in T2D sera that could be causing the increased number of CD8^+^ EMRA T cells (Figure 5C). While all inflammatory factors investigated were elevated, only C5, CXCL1, IL-6, IL-8, IL-16 and SerpinE1 were significantly upregulated. Both IL-6 and complement factor C5 are known to induce proliferation, expansion, and differentiation of T cells (Lalli et al., 2008; Li et al., 2018). Interestingly, C5, IL-8 and SerpinE1 have been demonstrated to be senescence inducing factors in human plasma irrespective of the senescent stimuli (Basisty et al., 2020). This suggests that T2D sera contains factors capable of driving premature senescence.

**FIGURE 6:**
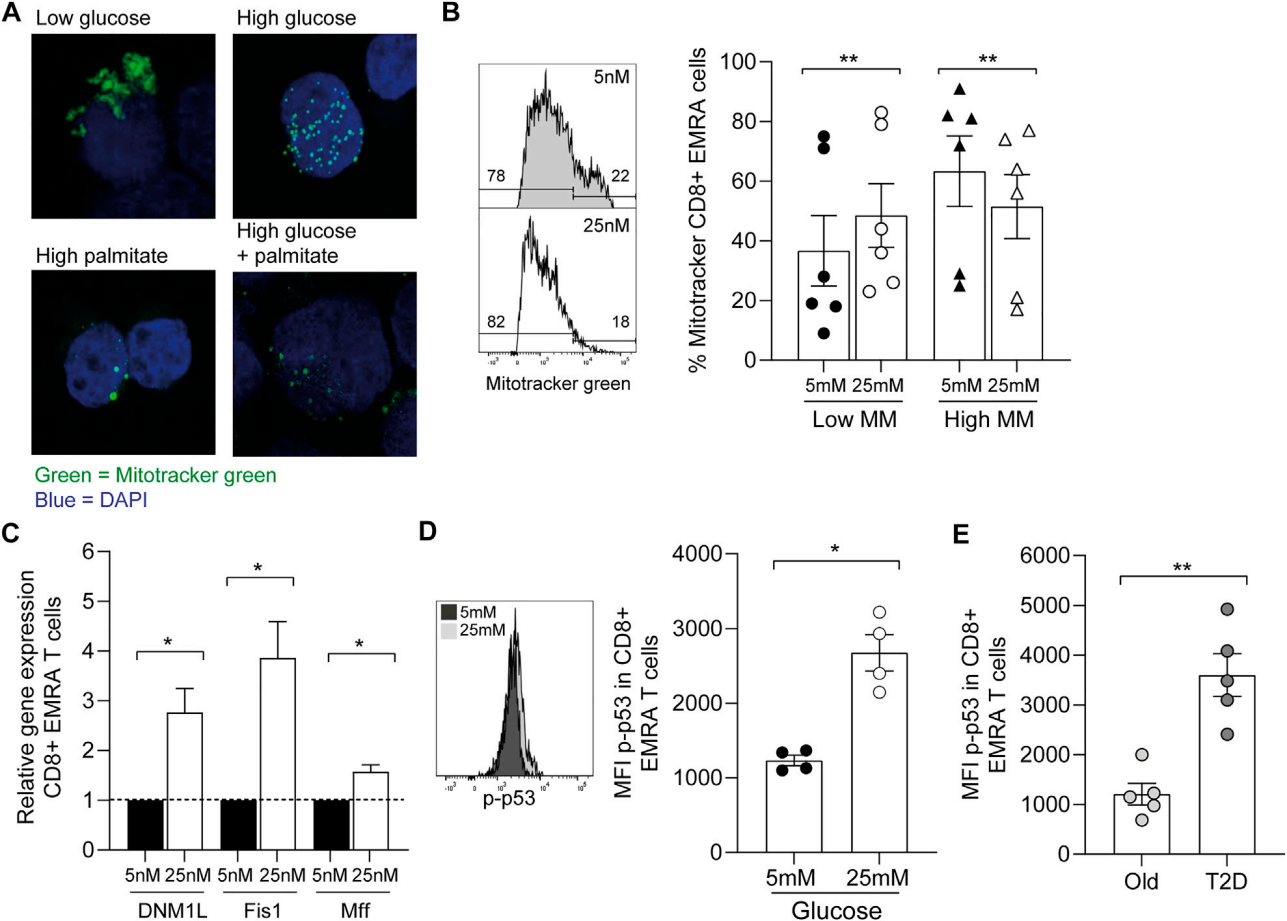
Increasing nutrient concentration leads to the fragmentation of mitochondria. **(A)** Representative images of Mitotracker green staining in CD8^+^ T cells incubated either in low glucose (5 mM), high glucose (25 mM), palmitate (200 μM) or both high glucose and palmitate. **(B)** Example and graph showing the mitochondrial mass of CD8^+^ EMRA T cells from young individuals incubated overnight with low or high glucose. *n* = 6. **(C)** qPCR data using CD8^+^ EMRA cells following mRNA levels of DNM1L, Fis1 and MffGATA3 following overnight incubation with low or high glucose, *n* = 3. All results are shown as ΔΔCT normalised against untreated samples. **(D)** Example and graph showing the expression of p-p53 in CD8^+^ EMRA T cells from young individuals following overnight incubation with low and high glucose. *n* = 4. **(E)** p-p53 expression from people living with T2D and age-matched controls. *n* = 5. All graphs show the mean ± SEM. *p* value was determined using a paired *t*-test, p* = < 0.05, p** = < 0.01.

While we found no change in T cell phenotype by increasing the concentrations of glucose and palmitate, we did observe more fragmentation of mitochondria (Figure 6A). These observed changes in mitochondrial morphology resemble the mitochondrial dysfunction seen *via* electron microscopy in CD8^+^ EMRA T cells taken from people living with T2D (Figure 2B). Indeed, when we incubated young PBMCs with low (5 mM) or high (25 mM) glucose we observe a shift to a lower mitochondrial mass in the CD8^+^ EMRA subset (Figure 6B). A lower mitochondrial mass can indicated the presence of smaller more fragmented mitochondria (Siska et al., 2017), and this was confirmed by qPCR. Transcription of genes associated with fission, *DNM1L*, *Fis1* and *Mff*, were found to be upregulated upon incubation with high glucose (Figure 6C). Furthermore, the changes observed using high glucose were associated with the induction of senescence, demonstrated by an increased expression of p-p53 in the CD8^+^ EMRA subset from young individuals (Figure 6D, Supplementary Figure S1E). This finding was mirrored in people living with T2D, where we observed a higher level of p-p53 expression compared to old age-matched controls (Figure 6E). Again this p-p53 data are suggestive of premature senescence in people living with T2D.

Taken together these data imply that the inflammatory serum factors observed in people living with T2D drives the differentiation of CD8^+^ T cells, the build-up of senescent CD8^+^ EMRA T cells leads to changes in nutrient uptake and usage promoting the accumulation of dysfunctional mitochondria. These poorly functioning mitochondria generate high levels of mtROS, further adding to the inflammatory burden in a detrimental feed-forward loop.

## Discussion

The impact of immunosenescence during ageing is well established, however it is now recognised to be a central finding in a plethora of syndromes including type 2 diabetes (Duncan et al., 2003). Immunosenescence is accompanied by alterations to T cell immunity and also by a low-grade chronic inflammatory state (Macaulay et al., 2013). We show here a rise in the presence of senescent CD8^+^ T cells in people living with T2D. Despite the use of medication to manage their disease, T2D participants exhibited greater levels of inflammatory mediators in their blood serum when compared to the age-matched control participants. It is important to note that it is hard to assess what the impact of these medications themselves are; however, it would be extremely challenging to find a large enough cohort of people living with T2D that did not manage their disease (or related conditions such as hypertension or hypercholesterolaemia) through medication to test this. Nevertheless, we found no correlation between the number of medications people were on and the number of CD8^+^ EMRA T cells. Furthermore, we found no effect of metformin usage on CD8^+^ EMRA T cells, a finding that is supported by work showing that thymocytes lack suitable transporters for its uptake (Vara-Ciruelos et al., 2019) and that metformin failed to impact major AMPK-sensitive metabolic pathways in PBMCs and CD4^+^ T cells (Nicholas et al., 2019). As a result, we find it unlikely the increase in CD8^+^ EMRA T cells is influenced by the medications used.

Inflammation plays a key role in the pathogenesis of many age-related and chronic diseases. Numerous studies have shown a correlation between elevated levels of inflammatory markers, such as IL-6 and C-reactive protein, and the risk of cardiovascular events (Kaptoge et al., 2012). TNF plays a crucial role in insulin resistance and the onset of T2D (Moller, 2000). Moreover, *in vitro* studies have shown that incubation with TNF is enough to drive the onset of T cell senescence. However, no TNF was detected in the sera of the T2D participants. We propose that this is due to both the short-lived nature of TNF and the fact that our study participants had been living with T2D for an average of 15 yr and were managing their disease through numerous medications. As a result, the chronic inflammatory cytokine signatures of these participants are likely to be different to the acute inflammatory cytokine signatures present during the onset of disease. However more inflammatory mediators were found in the sera of people living with T2D and these were able to differentiate CD8^+^ T cells to a greater extent than sera from age matched individuals. Additionally, a number of these serum factors, C5, SerpinE1 and IL-8, have also been shown to be part of the core senescence-inducing secretome (Basisty et al., 2020). However, further experiments are needed to fully appreciate the contribution of each cytokine to EMRA T cell generation.

Metabolic regulation plays an important role during T cell senescence, with mitochondrial dysfunction and elevated aerobic glycolysis now identified as key features of senescent CD8^+^ T cells (Henson et al., 2014). Since T2D is also characterised by metabolic imbalances we began to investigate T cell metabolism in the context of T2D. Mitochondria from T2D CD8^+^ T cells displayed a higher oxidative capacity, a phenomenon also observed in PBMCs isolated from people with T2D (Nicholas et al., 2017; Nicholas et al., 2019). This increased oxygen consumption from T2D CD8^+^ T cells did not equate to improved function, mitochondrial morphology showed T2D EMRA cells to be fragmented and the increased proton leak suggests more mitochondrial damage in T2D CD8^+^ T cells. mtROS was also elevated in T2D EMRA CD8^+^ T cells compared to healthy age-matched control cells. Oxidative stress in T2D is known to contribute to vascular complications such as atherosclerosis (Dandona et al., 1996; Baynes and Thorpe, 1999). Additionally, mtROS can also result in DNA damage and senescence (Davalli et al., 2016). Therefore, elevated mtROS levels in T2D CD8^+^ EMRA T cells could result in damage to the extracellular environment in which they sit and may also act to reinforce the senescent phenotype of these cells.

In activated T cells, insulin stimulates glucose uptake and enhances T cells responsiveness and energy requirements necessary for appropriate T cell function (Stentz and Kitabchi, 2003). It is therefore unsurprising that CD8^+^ EMRA T cells in the context of T2D, characterised by insulin resistance and deficiency, displayed alterations in glucose and fatty acid uptake. We show here that elevated levels of glucose and fatty acids are capable of generating the mitochondrial dysfunction seen during T2D. pAMPK, which is usually activated in response to low glucose and positively regulates FAO (Hardie, 2004; Xu et al., 2012), was reduced in T2D CD8^+^ EMRA T cells. Decreased AMPK could provide an explanation for the lack of FAO in these cells. Furthermore, if AMPK levels were increased in these cells FAO may be restored and the accumulation of FA in lipid droplets avoided. As glucose uptake and FAO are both impaired, it remains unclear what energy source T2D CD8^+^ T cells are metabolising. One potential candidate is glutamine. Glutamine mediated metabolism has been shown to be important during T cell activation and proliferation (Carr et al., 2010). It is used by many cell types in order to fuel oxidative metabolism under stress conditions (Yang et al., 2009; Le et al., 2012). Furthermore, a recent report has demonstrated a role for glutamine in maintaining the viability of senescent cells and the senescence-associated secretory phenotype (Johmura et al., 2021). However, as glutamine uptake is also positively regulated by pAMPK (Blagih et al., 2015), the reduced pAMPK levels in T2D EMRAs mean this hypothesis is unclear. To be certain, specific glutamine uptake assays need to be conducted.

In summary, the impaired nutrient uptake and usage in CD8^+^ EMRA T cells in T2D has a profound effect on their phenotype and function. Consequently, a better understanding of these metabolic abnormalities is crucial as metabolic manipulation of these cells may restore correct T cell function and help reduce the impact of T cell dysfunction in T2D.

## Data Availability

The original contributions presented in the study are included in the article/Supplementary Materials, further inquiries can be directed to the corresponding author/s.

## References

[B1] Acosta J. C. Banito A. Wuestefeld T. Georgilis A. Janich P. Morton J. P. (2013). A Complex Secretory Program Orchestrated by the Inflammasome Controls Paracrine Senescence. Nat. Cel Biol. 15, 978–990. 10.1038/ncb2784 PMC3732483 23770676

[B2] Alonso-Fernández P. De la Fuente M. (2011). Role of the Immune System in Aging and Longevity. Curr. Aging Sci. 4, 78–100. 10.2174/1874609811104020078 21235494

[B3] Antonescu C. N. McGraw T. E. Klip A. (2014). Reciprocal Regulation of Endocytosis and Metabolism. Cold Spring Harbor Perspect. Biol. 6, a016964. 10.1101/cshperspect.a016964 PMC4067987 24984778

[B4] Basisty N. Kale A. Jeon O. H. Kuehnemann C. Payne T. Rao C. (2020). A Proteomic Atlas of Senescence-Associated Secretomes for Aging Biomarker Development. Plos Biol. 18, e3000599. 10.1371/journal.pbio.3000599 31945054 PMC6964821

[B5] Baynes J. W. Thorpe S. R. (1999). Role of Oxidative Stress in Diabetic Complications: A New Perspective on an Old Paradigm. Diabetes 48, 1–9. 10.2337/diabetes.48.1.1 9892215

[B6] Blagih J. Coulombe F. Vincent E. E. Dupuy F. Galicia-Vázquez G. Yurchenko E. (2015). The Energy Sensor AMPK Regulates T Cell Metabolic Adaptation and Effector Responses In Vivo. Immunity 42, 41–54. 10.1016/j.immuni.2014.12.030 25607458

[B7] Bogdanski P. Pupek-Musialik D. Dytfeld J. Jagodzinski P. P. Jablecka A. Kujawa A. (2007). Influence of Insulin Therapy on Expression of Chemokine Receptor CCR5 and Selected Inflammatory Markers in Patients with Type 2 Diabetes Mellitus. Int. J. Clin. Pharmacol. 45, 563–567. 10.5414/cpp45563 17966842

[B9] Callender L. A. Carroll E. C. Bober E. A. Akbar A. N. Solito E. Henson S. M. (2020). Mitochondrial Mass Governs the Extent of Human T Cell Senescence. Aging Cell 19, e13067. 10.1111/acel.13067 31788930 PMC6996952

[B10] Carr E. L. Kelman A. Wu G. S. Gopaul R. Senkevitch E. Aghvanyan A. (2010). Glutamine Uptake and Metabolism Are Coordinately Regulated by ERK/MAPK during T Lymphocyte Activation. J. Immunol. 185, 1037–1044. 10.4049/jimmunol.0903586 20554958 PMC2897897

[B11] Chen F. Haigh S. Barman S. Fulton D. (2012). From Form to Function: The Role of Nox4 in the Cardiovascular System. Front. Physiol. 3, 412. 10.3389/fphys.2012.00412 23125837 PMC3485577

[B12] Chen S. de Craen A. J. Raz Y. Derhovanessian E. Vossen A. C. Rudi W. G. (2012). Cytomegalovirus Seropositivity Is Associated with Glucose Regulation in the Oldest Old. Results from the Leiden 85-plus Study. Immun. Ageing 9, 18. 10.1186/1742-4933-9-18 22929089 PMC3478991

[B13] Dandona P. Thusu K. Cook S. Snyder B. Makowski J. Armstrong D. (1996). Oxidative Damage to DNA in Diabetes Mellitus. Lancet 347, 444–445. 10.1016/s0140-6736(96)90013-6 8618487

[B14] Davalli P. Mitic T. Caporali A. Lauriola A. D'Arca D. (2016). ROS, Cell Senescence, and Novel Molecular Mechanisms in Aging and Age-Related Diseases. Oxid. Med. Cel Longev. 2016, 3565127. 10.1155/2016/3565127 PMC4877482 27247702

[B15] Desdín-Micó G. Soto-Heredero G. Mittelbrunn M. (2018). Mitochondrial Activity in T Cells. Mitochondrion 41, 51–57. 10.1016/j.mito.2017.10.006 29032101

[B16] Docherty M.-H. O’Sullivan E. D. Bonventre J. V. Ferenbach D. A. (2019). Cellular Senescence in the Kidney. J. Am. Soc. Nephrol. 30, 726–736. 10.1681/asn.2018121251 31000567 PMC6493983

[B17] Duncan B. B. Schmidt M. I. Pankow J. S. Ballantyne C. M. Couper D. Vigo A. (2003). Low-grade Systemic Inflammation and the Development of Type 2 Diabetes: The Atherosclerosis Risk in Communities Study. Diabetes 52, 1799–1805. 10.2337/diabetes.52.7.1799 12829649

[B18] Giubilato S. Liuzzo G. Brugaletta S. Pitocco D. Graziani F. Smaldone C. (2011). Expansion of CD4+CD28null T-Lymphocytes in Diabetic Patients: Exploring New Pathogenetic Mechanisms of Increased Cardiovascular Risk in Diabetes Mellitus. Eur. Heart J. 32, 1214–1226. 10.1093/eurheartj/ehq499 21217142

[B19] Goronzy J. J. Weyand C. M. (2019). Mechanisms Underlying T Cell Ageing. Nat. Rev. Immunol. 19, 573–583. 10.1038/s41577-019-0180-1 31186548 PMC7584388

[B20] Goronzy J. J. Weyand C. M. (2017). Successful and Maladaptive T Cell Aging. Immunity 46, 364–378. 10.1016/j.immuni.2017.03.010 28329703 PMC5433436

[B21] Hardie D. G. Ross F. A. Hawley S. A. (2012). AMPK: A Nutrient and Energy Sensor That Maintains Energy Homeostasis. Nat. Rev. Mol. Cel Biol. 13, 251–262. 10.1038/nrm3311 PMC5726489 22436748

[B22] Hardie D. G. (2004). The AMP-Activated Protein Kinase Pathway - New Players Upstream and Downstream. J. Cel Sci. 117, 5479–5487. 10.1242/jcs.01540 15509864

[B23] Henson S. M. Lanna A. Riddell N. E. Franzese O. Macaulay R. Griffiths S. J. (2014). p38 Signaling Inhibits mTORC1-independent Autophagy in Senescent Human CD8+ T Cells. J. Clin. Invest. 124, 4004–4016. 10.1172/jci75051 25083993 PMC4151208

[B24] Johmura Y. Yamanaka T. Omori S. Wang T.-W. Sugiura Y. Matsumoto M. (2021). Senolysis by Glutaminolysis Inhibition Ameliorates Various Age-Associated Disorders. Science 371, 265–270. 10.1126/science.abb5916 33446552

[B8] Kaptoge S. Di Angelantonio E. Pennells L. Wood A. M. White I. R. Gao P. (2012). C-Reactive Protein, Fibrinogen, and Cardiovascular Disease Prediction. N. Engl. J. Med. 367, 1310–1320. 10.1056/NEJMoa1107477 23034020 PMC3714101

[B25] Lalli P. N. Strainic M. G. Yang M. Lin F. Medof M. E. Heeger P. S. (2008). Locally Produced C5a Binds to T Cell-Expressed C5aR to Enhance Effector T-Cell Expansion by Limiting Antigen-Induced Apoptosis. Blood 112, 1759–1766. 10.1182/blood-2008-04-151068 18567839 PMC2518884

[B26] Lau E. Y. M. Carroll E. C. Callender L. A. Hood G. A. Berryman V. Pattrick M. (2019). Type 2 Diabetes Is Associated with the Accumulation of Senescent T Cells. Clin. Exp. Immunol. 197, 205–213. 10.1111/cei.13344 31251396 PMC6642873

[B27] Le A. Lane A. N. Hamaker M. Bose S. Gouw A. Barbi J. (2012). Glucose-independent Glutamine Metabolism via TCA Cycling for Proliferation and Survival in B Cells. Cel Metab. 15, 110–121. 10.1016/j.cmet.2011.12.009 PMC3345194 22225880

[B28] Lee Y.-h. Kim S. R. Han D. H. Yu H. T. Han Y. D. Kim J. H. (2019). Senescent T Cells Predict the Development of Hyperglycemia in Humans. Diabetes 68, 156–162. 10.2337/db17-1218 30389747

[B29] Li B. Jones L. L. Geiger T. L. (2018). IL-6 Promotes T Cell Proliferation and Expansion under Inflammatory Conditions in Association with Low-Level RORγt Expression. J. Immunol. 201, 2934–2946. 10.4049/jimmunol.1800016 30315140 PMC6324200

[B30] Lin S.-C. Hardie D. G. (2018). AMPK: Sensing Glucose as Well as Cellular Energy Status. Cel Metab. 27, 299–313. 10.1016/j.cmet.2017.10.009 29153408

[B31] Macaulay R. Akbar A. N. Henson S. M. (2013). The Role of the T Cell in Age-Related Inflammation. Age 35, 563–572. 10.1007/s11357-012-9381-2 22252437 PMC3636399

[B32] Mold J. E. Réu P. Olin A. Bernard S. Michaëlsson J. Rane S. (2019). Cell Generation Dynamics Underlying Naive T-Cell Homeostasis in Adult Humans. Plos Biol. 17, e3000383. 10.1371/journal.pbio.3000383 31661488 PMC6818757

[B33] Moller D. E. (2000). Potential Role of TNF-α in the Pathogenesis of Insulin Resistance and Type 2 Diabetes. Trends Endocrinol. Metab. 11, 212–217. 10.1016/s1043-2760(00)00272-1 10878750

[B34] Moura J. Madureira P. Leal E. C. Fonseca A. C. Carvalho E. (2019). Immune Aging in Diabetes and its Implications in Wound Healing. Clin. Immunol. 200, 43–54. 10.1016/j.clim.2019.02.002 30735729 PMC7322932

[B35] Nicholas D. A. Proctor E. A. Agrawal M. Belkina A. C. Van Nostrand S. C. Panneerseelan-Bharath L. (2019). Fatty Acid Metabolites Combine with Reduced β Oxidation to Activate Th17 Inflammation in Human Type 2 Diabetes. Cel Metab. 30, 447–461. 10.1016/j.cmet.2019.07.004 PMC8506657 31378464

[B36] Nicholas D. Proctor E. A. Raval F. M. Ip B. C. Habib C. Ritou E. (2017). Advances in the Quantification of Mitochondrial Function in Primary Human Immune Cells Through Extracellular Flux Analysis. PLoS One 12, e0170975. 10.1371/journal.pone.0170975 28178278 PMC5298256

[B37] Palmer A. K. Tchkonia T. LeBrasseur N. K. Chini E. N. Xu M. Kirkland J. L. (2015). Cellular Senescence in Type 2 Diabetes: A Therapeutic Opportunity. Diabetes 64, 2289–2298. 10.2337/db14-1820 26106186 PMC4477358

[B38] Patrick M. Weng N.-p. (2019). Expression and Regulation of Telomerase in Human T Cell Differentiation, Activation, Aging and Diseases. Cell Immunol. 345, 103989. 10.1016/j.cellimm.2019.103989 31558266 PMC6873926

[B39] Pereira B. I. De Maeyer R. P. H. Covre L. P. Nehar-Belaid D. Lanna A. Ward S. (2020). Sestrins Induce Natural Killer Function in Senescent-like CD8+ T Cells. Nat. Immunol. 21, 684–694. 10.1038/s41590-020-0643-3 32231301 PMC10249464

[B40] Plunkett F. J. Franzese O. Finney H. M. Fletcher J. M. Belaramani L. L. Salmon M. (2007). The Loss of Telomerase Activity in Highly Differentiated CD8+CD28−CD27−T Cells Is Associated with Decreased Akt (Ser473) Phosphorylation. J. Immunol. 178, 7710–7719. 10.4049/jimmunol.178.12.7710 17548608

[B41] Roy S. Leidal A. M. Ye J. Ronen S. M. Debnath J. (2017). Autophagy-Dependent Shuttling of TBC1D5 Controls Plasma Membrane Translocation of GLUT1 and Glucose Uptake. Mol. Cel. 67, 84–95. 10.1016/j.molcel.2017.05.020 PMC5522182 28602638

[B42] Shirakawa K. Yan X. Shinmura K. Endo J. Kataoka M. Katsumata Y. (2016). Obesity Accelerates T Cell Senescence in Murine Visceral Adipose Tissue. J. Clin. Invest. 126, 4626–4639. 10.1172/jci88606 27820698 PMC5127667

[B43] Siska P. J. Beckermann K. E. Mason F. M. Andrejeva G. Greenplate A. R. Sendor A. B. (2017). Mitochondrial Dysregulation and Glycolytic Insufficiency Functionally Impair CD8 T Cells Infiltrating Human Renal Cell Carcinoma. JCI Insight 2, e93411. 10.1172/jci.insight.93411 PMC5470888 28614802

[B44] Stegenga M. E. Crabben S. N. v. d. Dessing M. C. Pater J. M. van den Pangaart P. S. de Vos A. F. (2008). Effect of Acute Hyperglycaemia And/or Hyperinsulinaemia on Proinflammatory Gene Expression, Cytokine Production and Neutrophil Function in Humans. Diabetic Med. 25, 157–164. 10.1111/j.1464-5491.2007.02348.x 18290856 PMC2268957

[B45] Stentz F. Kitabchi A. (2003). Activated T Lymphocytes in Type 2 Diabetes: Implications from *In Vitro* Studies. Curr. Drug Targets 4, 493–503. 10.2174/1389450033490966 12866664

[B46] Vara-Ciruelos D. Dandapani M. Russell F. M. Grzes K. M. Atrih A. Foretz M. (2019). Phenformin, But Not Metformin, Delays Development of T Cell Acute Lymphoblastic Leukemia/Lymphoma via Cell-Autonomous AMPK Activation. Cel Rep. 27, 690–698. 10.1016/j.celrep.2019.03.067 PMC6484776 30995468

[B47] Velarde M. C. Flynn J. M. Day N. U. Melov S. Campisi J. (2012). Mitochondrial Oxidative Stress Caused by Sod2 Deficiency Promotes Cellular Senescence and Aging Phenotypes in the Skin. Aging 4, 3–12. 10.18632/aging.100423 22278880 PMC3292901

[B48] Wang Y. An H. Liu T. Qin C. Sesaki H. Guo S. (2019). Metformin Improves Mitochondrial Respiratory Activity Through Activation of AMPK. Cel Rep. 29, 1511–1523. 10.1016/j.celrep.2019.09.070 PMC6866677 31693892

[B49] Weyand C. M. Yang Z. Goronzy J. J. (2014). T-cell Aging in Rheumatoid Arthritis. Curr. Opin. Rheumatol. 26, 93–100. 10.1097/bor.0000000000000011 24296720 PMC3984035

[B50] Xu J. Ji J. Yan X.-H. (2012). Cross-talk Between AMPK and mTOR in Regulating Energy Balance. Crit. Rev. Food Sci. Nutr. 52, 373–381. 10.1080/10408398.2010.500245 22369257

[B51] Yang C. Sudderth J. Dang T. Bachoo R. G. McDonald J. G. DeBerardinis R. J. (2009). Glioblastoma Cells Require Glutamate Dehydrogenase to Survive Impairments of Glucose Metabolism or Akt Signaling. Cancer Res. 69, 7986–7993. 10.1158/0008-5472.can-09-2266 19826036 PMC2764330

[B52] Yu T. Robotham J. L. Yoon Y. (2006). Increased Production of Reactive Oxygen Species in Hyperglycemic Conditions Requires Dynamic Change of Mitochondrial Morphology. Proc. Natl. Acad. Sci. 103, 2653–2658. 10.1073/pnas.0511154103 16477035 PMC1413838

[B53] Zelle-Rieser C. Thangavadivel S. Biedermann R. Brunner A. Stoitzner P. Willenbacher E. (2016). T Cells in Multiple Myeloma Display Features of Exhaustion and Senescence at the Tumor Site. J. Hematol. Oncol. 9, 116. 10.1186/s13045-016-0345-3 27809856 PMC5093947

[B54] Zhu M. Liu X. Liu W. Lu Y. Cheng J. Chen Y. (2021). β Cell Aging and Age-Related Diabetes. Aging 13, 7691–7706. 10.18632/aging.202593 33686020 PMC7993693

