# Supplementary material for: Altered Nutrient Uptake Causes Mitochondrial Dysfunction in Senescent CD8+ EMRA T Cells During Type 2 Diabetes
Source: Front Aging. 2021 Aug 13;2:681428. doi: 10.3389/fragi.2021.681428 (PMC9261431; doi:10.3389/fragi.2021.681428)
Supplement: Supplementary file 2 [file Image1.pdf]

Supplementary Figure 1.

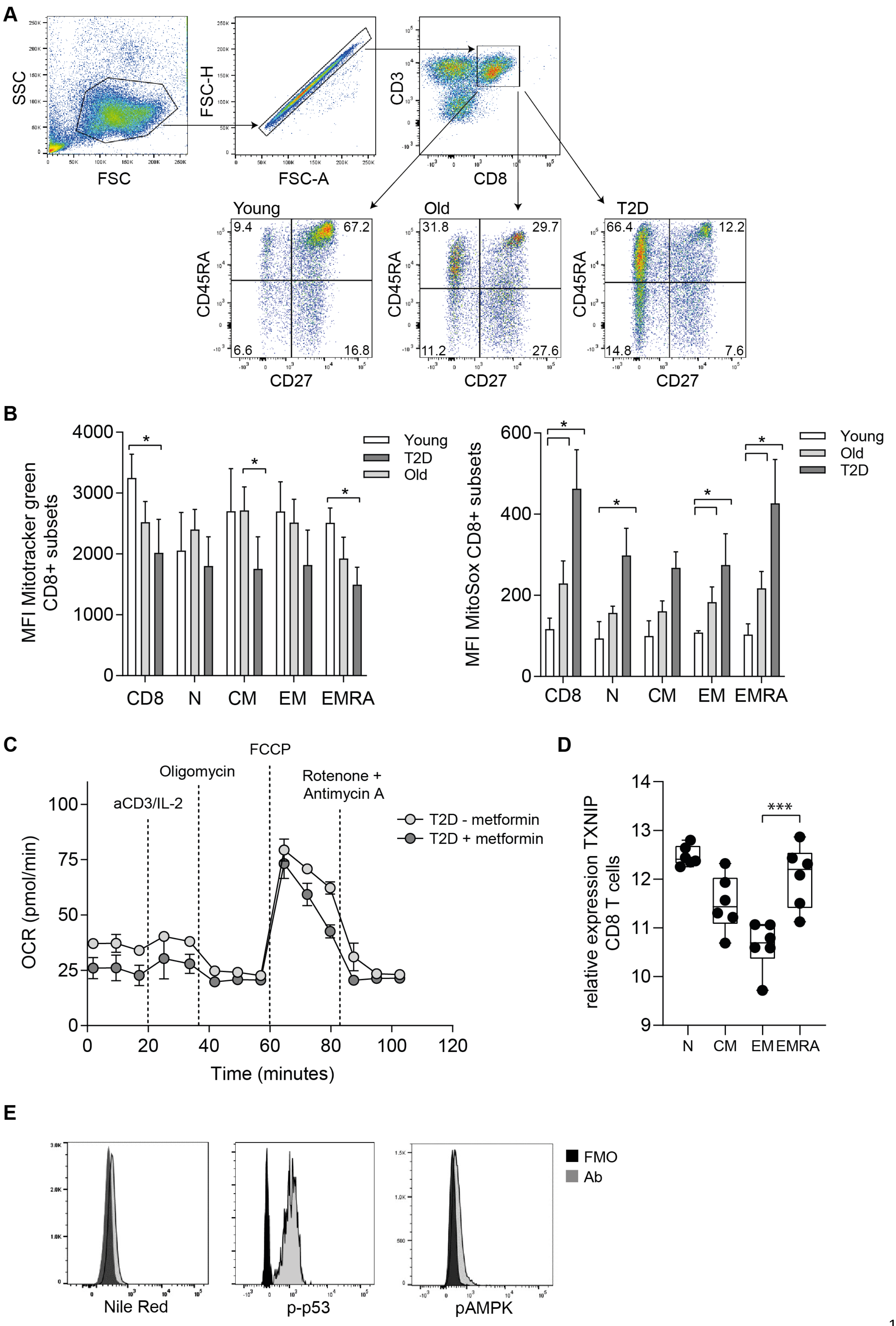

Supplementary Figure 1. Phenotyping of CD8$^+$ EMRA T cells

(A) Gating strategy for CD27/CD45RA defined CD8$^+$ T cell subsets. From left to right, lymphocytes, single cells, CD3$^+$CD8$^+$ T cells and CD27/CD45RA subsets from young, old and people living with T2D. (B) Quantification of Mitotracker green and MitoSOX in total CD8$^+$ T cells together with CD27/CD45RA defined CD8$^+$ T cell EMRAs isolated from young, older individuals and people living with T2D. Data expressed as mean ± SEM, n = 7. (C) Oxygen consumption rates (OCR) of CD8$^+$ T cells from people living with T2D who received metformin compared to those who did not. (D) Relative expression of thioredoxin-interacting protein using the deposited data set GEO Series accession number GSE98640 from 6 old individuals. (E) Examples of Nile red, pAMPK and p-p53 staining with and without an FMO control used to set the positive and negative gate. P values were determined using a two-way ANOVA with Bonferroni analysis used for post-hoc testing, p* = < 0.05.
